# Removing nucleic acids from nucleoid-associated proteins purified by affinity column

**DOI:** 10.14440/jbm.2016.98

**Published:** 2016-01-30

**Authors:** Audrey Vingadassalon, Philippe Bouloc, Sylvie Rimsky

**Affiliations:** ^1^LBPA, ENS Cachan, CNRS, Université Paris-Saclay, F-94235 Cachan, France; ^2^Institute for Integrative Biology of the Cell (I2BC), CEA, CNRS, Université Paris-Sud, Université Paris-Saclay, F-91198, Gif-sur-Yvette cedex, France

**Keywords:** affinity, nucleic acids, nucleoid-associated proteins, purification

## Abstract

In bacteria, DNA is tightly compacted in a supercoiled organization, which is mediated in part by nucleoid-associated proteins (NAPs). NAPs are well characterized for their ability to bind nucleic acids and for their involvement in gene regulation. A method commonly used to study protein-nucleic acid interactions involves immunoprecipitation of the protein of interest which is subsequently incubated with nucleic acids. A common cause of artifact is due to nucleic acids that remains bound to the protein of interest during the whole purification process. We developed an optimized method for the purification of tagged NAPs on affinity columns. The combination of three known methods allows removal of most of the nucleic acids bound to proteins during the purification process. This protocol is designed to improve the quality and specificity of results of *in vitro* experiments involving nucleic acid binding tests on purified NAPs. It can be used for *in vitro* studies of other RNA/DNA binding proteins.

## BACKGROUND

In bacteria, DNA is tightly condensed in a structure called nucleoid and closely associated with structuring proteins such as polymerases, topoisomerases and nucleoid-associated proteins (NAPs) [1]. The nucleoid presents several organization levels. The first level is a 10 nm fiber composed of DNA and nucleoid proteins, and further folding results in the second level in which a 30 nm fiber is formed due to the presence RNA molecules [2].

In *Escherichia coli* K12, the most abundant NAPs are Fis, IHF, HU, Dps, and H-NS and its paralog StpA [3]. All those proteins share the properties of binding and compacting DNA by bending, wrapping or bridging it. As such, NAPs play an important role in the maintenance of a dynamic genome and directly or indirectly control gene expression [4,5]. A few reports also point to the association of NAPs with RNA [6–9]. Their mode of interaction with the nucleic acids is variable and sequence specificity ranges from low to high [3].

Deep-sequencing-based experiments including whole-genome sequencing to identify the targets of DNA structural proteins are increasingly utilized in functional genomics. A method commonly used to study *in vitro* interactions between proteins and nucleic acids is coimmunoprecipitation (Co-IP) followed by high-throughput sequencing [10]. The first step of this method consists in overproducing tagged proteins followed by their purification on affinity columns. Then, purified proteins can be used for *in vitro* binding experiments with nucleic acids. However, a limit of this procedure is the presence of nucleic acids which remain associated to NAPs despite the purification steps. Experiments carried out in our laboratory revealed that nucleic acids from the producer strain were bound to NAPs of interest and consequently alter the interpretation of Co-IP experiments.

We combined three known biochemistry and molecular biology methods to improve NAP purification. A Benzonase nuclease treatment, an ammonium sulfate precipitation and a heparin affinity chromatography were associated to remove nucleic acids bound to NAPs, e.g., H-NS and StpA.

## MATERIALS

### Reagents

• Acetic acid (VWR, cat. # 20104.298)
• Ammonium sulfate (Merck, cat. # 1012171000)
• Ampicillin (Sigma-Aldrich, cat. # A9518)
• Anti-Flag M2 affinity gel (Sigma-Aldrich, cat. # A2220)
• Bacterial strain for protein expression BL21(DE3) (Invitrogen)
• Benzonase nuclease (Novagen, cat. # 70664-3)
• Circular plasmid DNA pET29b (Novagen, cat. # 69872-3)
• Cloning vector pET21a (+) (Novagen, cat. # 69740-3)
• Di-sodium hydrogen phosphate (Na_2_HPO_4_.2H_2_O, cat. # Merck 1065861000)
• DreamTaq DNA polymerase (Thermo Scientific, cat. # EP0711)
• Ethanol absolute (VWR, cat. # 20820.293)
• Ethylenediaminetetraacetic acid (EDTA, Sigma-Aldrich, cat. # E5134)
• GeneRuler DNA ladder mix (Thermo Scientific, cat. # SM0331)
• Glycerol (VWR, cat. # 24388.295)
• Glycine (Sigma-Aldrich, cat. # G8898)
• HiTrap heparin HP 1 ml (GE Healthcare, cat. # 17-0406-01)
• Isopropyl β-D-1-thiogalactopyranoside (ITPG, Sigma-Aldrich, cat. # I6758)
• LD-Dithiothreitol (DTT, Sigma-Aldrich, cat. # D0632)
• Lennox broth medium (Fischer scientific, cat. # BP1427)
• PageRuler prestained protein ladder (Thermo Scientific, cat. # 26616)
• PlusOne Coomassie tablets PhastGel Blue R-250 (GE Healthcare, cat. # 17-0518-01)
• Protease inhibitor cocktail (Sigma-Aldrich, cat. # P8465)
• Reagents for standard SDS-PAGE assay
• Sodium chloride (Merck, cat. # 106404-1000)
• Sodium dihydrogen phosphate (NaH_2_PO_4_.2H_2_O, Merck, cat. # 1063461000)
• SuperScript III reverse transcriptase (Invitrogen, cat. # 18080-044)
• Trizma base (Sigma-Aldrich, cat. # T1503)
• TurboDNase (Ambion, cat. # AM2238)

### Equipment

• AKTA purifier Box 900 system including pump P-900, monitor UV-900 and monitor pH/C900 (Amersham Pharmacia Biotech)
• Bio-Spin disposable chromatography columns (Bio-Rad, cat. # 732-6008)
• Dialysis tubing Nominal filter 6K MWCO (Roth, cat. # E668.1)
• Standard equipment SDS-PAGE assay
• French press
• Mixed cellulose esters membrane 0.22 µM (Millipore, cat. # GSWP04700)
• Mortar
• Refrigerated centrifuge
• Slide-A-Lyzer™ dialysis cassettes, 10K MWCO, 3 ml (Thermo Scientific, cat. # 66380)
• Standard cell culture equipment

### Buffers

• Buffer A: 50 mM Tris-HCl, 2 mM EDTA, 10% glycerol, pH 7.4
• Buffer B: 10 mM NaHPO_4_, 100 mM NaCl, 2 mM EDTA, 5% glycerol, pH 7.4
• Buffer C: 10 mM NaHPO_4_, 100 mM NaCl, 5% glycerol, pH 7.4
• Buffer D: 10 mM NaHPO_4_, 1 M NaCl, 5% glycerol, pH 7.4
• Buffer E: 50 mM Tris-HCl, 150 mM NaCl, 5% glycerol, pH 7.4
• Buffer F: 500 mM Tris-HCl, 1.5 M NaCl, pH 7.4
• Storage buffer: 50% glycerol, 25 mM Hepes (pH 7.4), 0.05% NP40, 300 mM NaCl
**CAUTION**: For H-NS purification, all the buffers are supplemented with 4 mM DTT.

## PROCEDURE

### Protein extract preparation

**NOTE**: All buffers are filtered on 0.22 µM cellulose membranes and precooled at 4°C. SDS-PAGE gels are stained with Coomassie blue R-250.
1. Streak the strain of interest on Lennox Broth (LB) agar plate supplemented with the appropriate antibiotic. Incubate overnight at 37°C.
2. Inoculate 10 ml of LB supplemented with the appropriate antibiotic with an isolated colony. Incubate overnight at 37°C under 180 rpm shaking.
3. Inoculate 500 ml of LB supplemented with the appropriate antibiotic with 5 ml of the overnight culture. Incubate at 37°C under 180 rpm shaking until OD_600_ = 0.3. Remove a 2-ml aliquot and store it on ice for further SDS-PAGE analysis.
4. Induce with the appropriate inducer. Incubate 1 h at 37°C under 180 rpm shaking. Remove a 2 ml aliquot and store it on ice for further SDS-PAGE analysis.
5. Centrifuge the culture at 3000 g for 10 min at 4°C and discard the supernatant.
6. Wash the pellet with 250 ml of buffer A.
7. Centrifuge the culture at 3000 g for 10 min at 4°C and discard the supernatant.
8. Repeat twice steps 6 and 7 with 100 ml and then 50 ml of buffer A.
9. Freeze the pellet with liquid nitrogen. The pellet can be stored at -80°C.
**Note**: Optional step allowing sample storage and improving cell lysis.
10. Thaw the pellet on ice. Resuspend it very carefully with 9 ml of buffer A. Add 1 ml of protease inhibitor cocktail. Mix gently.
11. Lyse bacteria with 3 cell disruption cycles using a French Press (under a 10000 psi pressure) or equivalent equipment.
**Note**: Three cycles are recommended to insure efficient cell lysis and DNA shearing. The solution should be clear and not viscous at the end of the process.
**CAUTION**: Keep the sample as cool as possible during this process.
12. Keep the processed samples on ice.
13. Add 2 mM MgCl_2_ and 50 units of benzonase nuclease. Mix gently. Incubate 30 min at 37°C.
**CAUTION:** Formation of foam indicates protein denaturation.
14. Add 4 units of TurboDNase. Incubate 30 min at 37°C.
15. Centrifuge the protein extract for 1 h at 20000 g at 4°C and recover the supernatant. Remove a 20 µl aliquot and store on ice for further SDS-PAGE analysis, benzonase activity test and nucleic acids contamination test.

### Ammonium sulfate precipitation

**NOTE**: Protein precipitation is performed by adding progressively ammonium sulfate from 0 to 40% then 40 to 60% for H-NS, 0 to 60% for StpA.
16. Adjust the volume (step 15) to 20 ml with buffer A.
17. Add 0.5 M NaCl.
18. Put the tube in a beaker containing ice. Mix the solution via a magnetic stirrer.
19. Weigh ammonium sulfate to bring the solution to the expected final concentration and crush it until getting a fine powder.
20. While the sample is stirring, slowly add ammonium sulfate and check the pH regularly.
**CAUTION**: The stirring has to be gentle to avoid foaming (protein denaturation). The pH may increase by adding ammonium sulfate; in this case, it should be adjusted to 7.4 with 1M HCl.
21. Once ammonium sulfate is added and dissolved, incubate at least 30 min at 4°C.
22. Centrifuge at 27000 g 30 min at 4°C and pick up the pellet.
**CAUTION**: Keep the supernatant at 4°C for a control at the end of the process in case the protein is not found.
23. Resuspend the pellet in 5 ml of buffer B.
24. Transfer the sample in a dialysis tube.
25. Fill a 1 L cylinder with 1 L of buffer B.
26. Put a magnetic stir bar, and immerse the dialysis tube containing the sample in buffer B. Place the graduated cylinder on a magnetic plate in a cold room and stir the solution.
27. After 1 h, replace the buffer with fresh buffer B. Stir overnight.
**NOTE**: Since the sample is resuspended in the buffer B, it is also dialyzed against buffer B to remove co-precipitated ammonium sulfate and sodium chloride before purification on heparin column.

### Purification on heparin column

28. Recover the sample from the dialysis tube.
29. Centrifuge at 4500 g for 15 min at 4°C (to remove any precipitate that can occur during dialysis and interfere with the FPLC process). Remove a 20 µl aliquot for further SDS-PAGE analysis.
30. Equilibrate a 1 ml heparin column with 20 volumes of buffer C at a flow rate of 1 ml/min. Load the sample on the heparin column at a flow rate of 0.3 ml/min.
31. Wash the column with 20 volumes of buffer C at a flow rate of 1 ml/min. Check the OD at the exit of the column: it should be back to the background buffer-absorption before to proceed to the next step.
32. Run an elution gradient from 0 to 100% of heparin buffer D in 30 min at a flow rate of 0.3ml/min and collect 300 µl fractions. Remove and keep a 10 µl aliquot from each fraction for further SDS-PAGE analysis.
**NOTE**: During the gradient, the salt concentration in the heparin column will increase and allow protein elution.
**NOTE**: The column can be reused. Wash it with 10 column volumes of buffer D and make a 2 ml gradient from 100% to 0% buffer C. Finally, wash it again with 10 column volumes of buffer C. It can then be reloaded or stored by washing with 10 column volumes of 20% Et-OH (for flow rate, follow the manufacturer instructions).
**TIP**: Run an SDS-PAGE gel with all the fractions collected since the beginning. Crude extracts from steps 3 and 4 have to be diluted ten times before loading on the gel. 10 µl of each sample after centrifugation (step 29) and of the elution fractions (step 32) are directly loaded. Tips for troubleshooting can be found in table 1.

### Purification on Anti-Flag M2 affinity gel

33. Pool all fractions containing the protein of interest and transfer the sample (around 1.5 ml) in a 3 ml dialysis cassette.
34. Fill a 1 L beaker with 1 L of dialysis buffer E.
35. Put a magnetic stir bar in the beaker and immerse the dialysis cassette. Then leave the beaker in the cold room with stirring.
36. After 1 h, replace the buffer with fresh buffer E. Stir overnight.
37. Recover the sample from the dialysis cassette. Remove a 10 µl aliquot for SDS-PAGE analysis.
38. Centrifuge at 4500 g for 15 min at 4°C (same note as step 29).
39. Rinse an empty 2 ml Bio-Rad microspin column with 2 column volumes of 0.1 M glycine, pH 3.5, then with 2 volumes of buffer E with gravity flow rate.
40. Load the Anti-Flag M2 affinity gel (for volume, follow the manufacturer instructions).
41. Rinse the resin three times with one column volume of 0.1 M glycine, pH 3.5.
42. Equilibrate the resin five times with one column volume of buffer E.
43. Load the sample on the resin with gravity flow rate. Collect the flow-through and reload the resin five times successively with gravity flow rate.
44. Wash the resin five times with one column volume of buffer E.
45. Elute the protein with five column volumes of 0.1 M glycine, pH 3.5; Collect 450 µl fractions in tubes already containing 50 µl of buffer F.
46. Pool the fractions containing the protein of interest and transfer the sample (around 1.5 ml) in a 3 ml dialysis cassette.
47. Fill a 1 L beaker with 1 L of storage buffer.
48. Put a magnetic stir bar in the beaker and immerse the dialysis cassette. Then leave the beaker in the cold room with stirring.
49. After dialysis for 1 h, replace the buffer with fresh storage buffer. Stir overnight.
50. Recover the sample from the dialysis cassette (around 1.5 ml). Centrifuge at 4500 g for 15 min at 4°C (same note as step 29). Remove a 20 µl aliquot and store on ice for further SDS-PAGE analysis and nucleic acids contamination test. Store the purified protein at -20°C.
**TIP**: Run an SDS-PAGE gel with 10 µl of each sample after centrifugation (step 38) and 10 µl of the elution fractions (step 45).
**CAUTION**: Don’t forget to supplement the storage buffer with 4 mM DTT for H-NS.

## ANTICIPATED RESULTS

**Figure 1.**
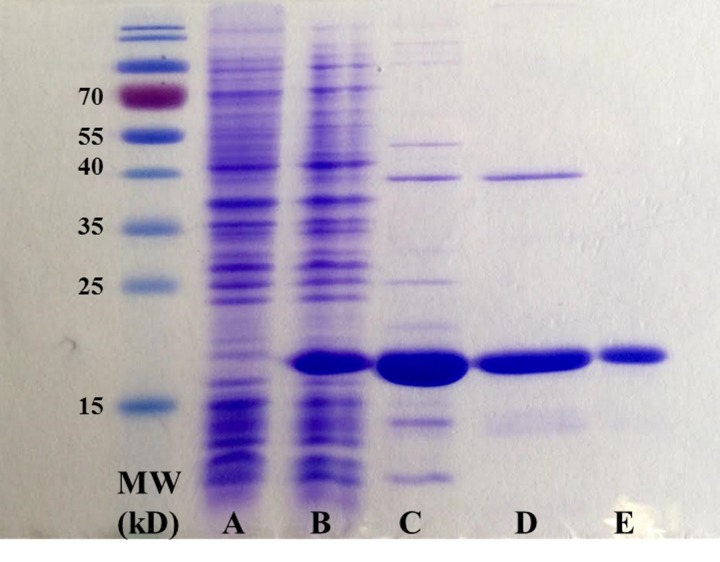
**SDS-PAGE analysis of H-NS-3xF (clone 1) protein extracts. A. Before IPTG induction (step 3, 5 µl), B.** After 1 h IPTG induction (step 4, 5 µl), **C.** Final pellet after ammonium sulfate precipitation (step 29, 5 µl), **D.** Pool of fractions containing H-NS-3xF eluted from the heparin column (step 37, 5 µl), **E.** Pool of fractions containing H-NS-3xF eluted from the Anti-FLAG resin (step 50, 5 µl). MW, molecular weight ladder (5 µl).

**Figure 2.**
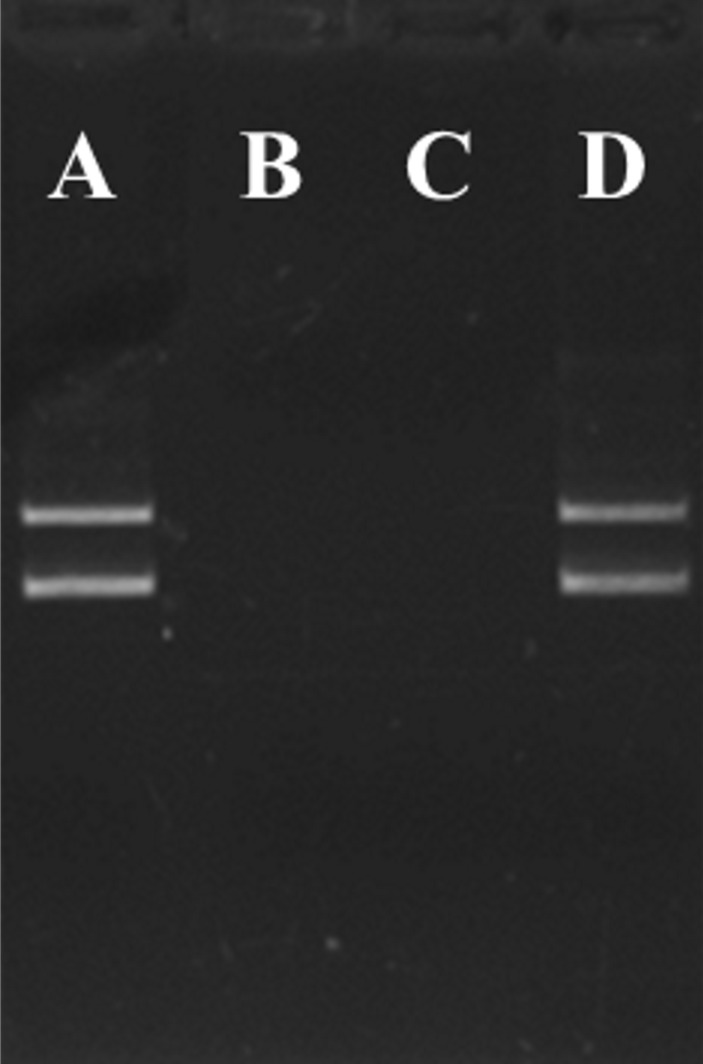
**Benzonase nuclease activity test**. Circular pET29b plasmid DNA (150 ng) was incubated 1 h at 37°C, and then loaded on a 1% agarose gel. Incubation with: **A.** Buffer A supplemented with 2 mM MgCl_2_, **B.** H-NS protein extract after heparin column purification, **C.** 3 µl of flow-through after loading of H-NS protein extract on Anti-flag M2 resin or D. Purified H-NS protein.

**Figure 3.**
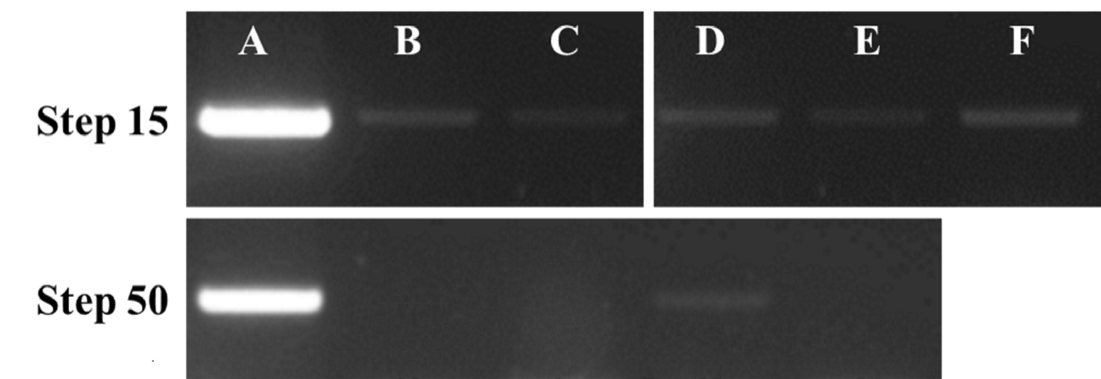
DNA contamination test. PCR amplification to detect a 160 nt fragment of *gcvB* was performed on BL21 genomic DNA and protein extracts from step 15 (upper) and step 50 (lower) of the 3-step procedure. **A.** BL21 DNA positive control. **B.** H-NS clone 1, **C.** H-NS clone 2, **D.** StpA clone 1, **E.** StpA clone 2 or **F.** H-NS sample purified by classical affinity column method.

**Figure 4.**
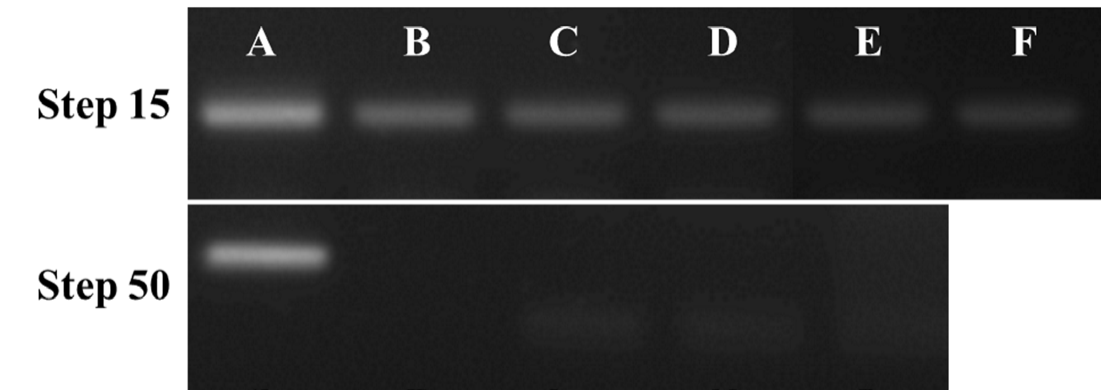
**RNA contamination test**. RT-PCR experiments to detect a 160 nt fragment of GcvB RNA were performed on BL21 total RNA and protein extracts from different steps of the 3-step procedure after nuclease treatment (step 15) and at the end of the procedure (step 50) as indicated. **A.** BL21 total RNA prior to treatment. **B.** H-NS clone 1, **C.** H-NS clone 2, **D.** StpA clone 1, **E.** StpA clone 2 or **F.** H-NS sample purified by classical affinity column method.

The protocol concerns the purification of two fusion NAPs with a C-terminal 3xFlag tail: H-NS-3xF (18 kDa) and StpA-3xF (18 kDa). Their corresponding genes were cloned in vector pET21 and expressed in strain *E. coli* BL21 (DE3). The proposed method adds three steps to the standard NAP purification by affinity columns: i) benzonase nuclease treatment, ii) ammonium sulfate precipitation and iii) heparin column purification. The current 3-step procedure was used to purify biological duplicates of each protein (**Fig. 1-4** and data not shown).

The purification efficiency was monitored at different steps of the procedure. SDS-PAGE analysis of different samples taken from the H-NS-3xF replicate 1 purification is shown in **Figure 1**: H-NS was shown to be pure at the end of the procedure (**Fig. 1E**). Similar results were obtained for the second replicate of H-NS and the StpA samples (data not shown).

As the method includes benzonase treatment, the presence of the nuclease was monitored during the purification procedure. 150 ng of a pET29b circular plasmid DNA (**Fig. 2A**) was incubated 1 h at 37°C with protein extracts from different steps of the purification (**Fig. 2**). The plasmid was added in a 20 µl total mix containing the same concentration of protein extract (estimated on Coomassie blue SDS-PAGE) and 2 mM MgCl2 as required for benzonase activity. The results showed that the nuclease is still active after the heparin purification as the plasmid was digested (**Fig. 2B**). However, at the final step of protein purification on the anti-Flag M2 resin, the nuclease was removed from the protein and found in the flow-through. The plasmid DNA is digested by the recovered flow-through (**Fig. 2C**) but not by the eluted protein (**Fig. 2D**).

Another important step in this procedure is purification by a column containing heparin sepharose. This affinity chromatography column is commonly used for protein purification to remove bound nucleic acids.

Efficiency of the procedure was tested by PCR for the presence of DNA and by reverse transcription polymerase chain reaction (RT-PCR) for the presence of RNA. PCR amplifications were performed with the DreamTaq DNA polymerase according to manufacturer’s instructions. RT-PCRs were performed using SuperScript III RT according to manufacturer’s instructions; the obtained complementary DNA strands were then used as template for PCR-amplifications with 30 cycles.

Previous tests carried out in our laboratory suggested that H-NS and StpA bind to GcvB (unpublished data). Consequently, we performed PCRs and RT-PCRs using gcvB_F (TTATCGGAATGCGTGTTCTG) and gcvB_R (TAGGCGGTGCTACATTAATC) primers amplifying a 160-nt fragment. BL21 genomic DNA and RNA extracts were used as a control for the PCR and RT-PCR reactions, respectively (**Fig. 3A** and **4A**). An H-NS sample treated with DNase and then purified with a classical affinity column method was also tested (**Fig. 3F** and **4F**). Experiments revealed that remaining gcvB DNA and GcvB RNA are bound to H-NS when purified only using the Anti-Flag M2 resin and following manufacturer’s instructions.

The current 3-step procedure was used to purify tagged H-NS and StpA proteins in duplicate (clone 1 and 2). PCRs and RT-PCRs were performed on the four samples after nuclease treatment (step 15). The PCR results obtained for both H-NS (**Fig. 3B** and **3C**, step 15) and StpA (**Fig. 3D** and **3E**, step 15) showed weak signals, indicating that gcvB DNA is still bound to the proteins. Moreover, weak signals obtained by RT-PCRs revealed that GcvB RNA is still bound to both proteins (**Fig. 4**, step 15).

At the end of the 3-step purification (step 50), PCRs and RTPCRs were performed on H-NS and StpA samples (**Fig. 3** and 4, step 50). We use as template an equal protein concentration (estimated by Coomassie blue SDS-PAGE) as for the PCRs and RT-PCRs run at step 15. No significant bands were detected, as tested by PCR (**Fig. 3**, step 50) and by RT-PCR (**Fig. 4**, step 50) in H-NS (**Fig. 3B** and **3C**, **4B** and **4C**, step 50) and StpA (**Fig. 3D** and **3E**, **4D** and **4E**, step 50) samples. A slight band corresponding to gcvB DNA is still visible in the StpA “clone 1” sample (**Fig.** 3D, step 50). However, during the following Co-IP experiment, the DNase treatment step removed this remaining DNA.

We propose here an optimized method to efficiently purify tagged NAPs by removing most bound nucleic acids. The protocol will facilitate *in vitro* studies of NAPs. It can also be adapted to other proteins with nucleic acids binding properties such as RNA chaperones (*e.g.*, Hfq), topoisomerases or polymerases.

**Table 1.** Troubleshooting.

Step	Problem	Possible reasons	Solution
13	No benzonase nuclease activity	Incompatible buffer	Check buffer concentrations for Mg^2+^ (at least 1 mM), EDTA (< 1 mM)
15	Protein detectable in the pellet after centrifugation	• Protein aggregation	• Performed induction at 16^°^C during 16 h
		• Protein in inclusion bodies	• Reduce inducer concentration.
29	No protein detectable in the pellet after ammonium sulfate precipitation	• Failed precipitation	• Check stirring and pH variation during ammonium sulfate addition. Incubate precipitation mix longer than 30 min (1 to 3 h).
		• Excess of glycerol in the buffer	• Dilution prior centrifugation.

## Abbreviations used

NAP: Nucleoid-associated protein
H-NS: Nucleoid structuring protein
Co-IP: co-immunoprecipitation
